# Oxidative Stress and Cytoskeletal Reorganization in Hypertensive Erythrocytes

**DOI:** 10.3390/antiox14010005

**Published:** 2024-12-24

**Authors:** Ivette Martínez-Vieyra, Isaac Hernández-Rojo, Víctor Hugo Rosales-García, Aracely Evangelina Chávez-Piña, Doris Cerecedo

**Affiliations:** 1 Laboratorio de Hematobiología, Escuela Nacional de Medicina y Homeopatía, Instituto Politécnico Nacional, Mexico City 07700, Mexico; iamartinez@ipn.mx (I.M.-V.); ihernandezr1405@alumno.ipn.mx (I.H.-R.); 2 Laboratorios Centrales, Centro de Investigación y de Estudios Avanzados del IPN, Mexico City 07360, Mexico; vrosales@cinvestav.mx; 3 Laboratorio de Farmacología, Escuela Nacional de Medicina y Homeopatía, Instituto Politécnico Nacional, Mexico City 07700, Mexico; achavezp@ipn.mx

**Keywords:** red blood cells, reactive oxygen species, protein modification, cytoskeleton reorganization, erythrocytes heterogeneity

## Abstract

Oxidative stress is widely recognized as a key mechanism in the development of hypertension. Under pathological conditions, such as in hypertension, oxidative stress leads to irreversible posttranslational modifications of proteins, which result in loss of protein function and cellular damage. We have previously documented physiological and morphological changes across various blood and bone marrow cell lineages, all of which exhibit elevated oxidative stress. While cytoskeletal changes in erythrocytes have been well characterized in hereditary diseases, this is the first study, to our knowledge, to investigate cytoskeletal reorganization in erythrocytes from hypertensive patients. To this end, we compared the expression patterns and subcellular distribution of key cytoskeletal proteins in erythrocytes from hypertensive individuals with those from normotensive subjects using Western blot, flow cytometry, and confocal microscopy. Our results revealed the presence of three erythrocyte subpopulations with differential expression of glycophorin A. The persistent oxidative environment in hypertensive patients causes dysregulation in the expression of glycophorin A, Band 3 protein, protein 4.1, and ankyrin, as well as the reorganization of spectrin. These alterations in protein expression and distribution suggest that oxidative stress in hypertensive individuals may induce structural modifications, ultimately impairing erythrocyte membrane elasticity and function.

## 1. Introduction

Arterial hypertension (HTN) is recognized as the leading global risk factor for mortality among non-communicable diseases [1]. HTN is a multifactorial condition with an unclear etiology, and its pathogenesis is associated with redox imbalances, specifically between elevated production of reactive oxygen species (ROS)—such as superoxide, hydrogen peroxide, and hydroxyl radical—and/or reduced antioxidant capacity, both systemically and locally within the circulatory system [2,3]. ROS are known to exert both physiological and pathophysiological roles, depending on their concentrations and subcellular localization [4]. In controlled levels, ROS contribute to cellular signaling and phenotype regulation. However, excessive ROS production disrupts critical pathways that regulate vascular resistance and blood pressure, including reduced nitric oxide (NO) bioavailability, increased inflammation, imbalances in salt and water homeostasis, hyperactivation of the sympathetic nervous system (SNS), and dysregulation of the renin-angiotensin-aldosterone system (RAAS) [5].

Oxidative stress occurs when the balance between elevated ROS and reactive nitrogen species (RNS), which are byproducts of cellular metabolism, and the antioxidant defense capacity is disrupted. ROS and RNS are continuously neutralized by a variety of intra- and extracellular antioxidant mechanisms. Erythrocytes, which are constantly exposed to ROS and RNS during circulation, rely heavily on hemoglobin (Hb) for redox reactions. In addition, erythrocytes contain a range of enzymatic antioxidants, including peroxiredoxin II (PRDX2), glutathione peroxidase (Gpx), and catalase, as well as low-molecular-weight antioxidants such as α-tocopherol, ascorbate, and the glutathione (GSH) system, which prevent lipid oxidation. These antioxidant systems are fueled by NADPH, primarily generated through the pentose phosphate pathway [6].

It has long been considered that the primary function of erythrocytes is to deliver O₂ to body tissues. However, the concept of the reactive species interactome has recently been introduced to describe the complex redox biology and physiology of erythrocytes. This interactome is defined as an “oxidation-reduction system” consisting of chemical interactions between reactive sulfur species (RSS), reactive nitrogen species (RNS), and reactive oxygen species (ROS) with their thiol targets. This concept, elaborated in detail by Miriam M. Cortese-Krott in 2023 [7], highlights the pivotal role of hemoglobin and its various redox forms, such as the reduced forms (oxyHb and deoxyHb) and the oxidized form (metHb). The latter serves as a primary source of superoxide and other oxidants, which are subsequently neutralized by antioxidant systems. Moreover, oxyHb and deoxyHb are actively involved in nitric oxide (NO) metabolism, generating HbNO and producing NO from nitrite. As a result, erythrocytes play an active role in the systemic regulation of NO metabolism and bioactivity. Additionally, metHb forms a stable adduct with sulfide, which can be converted back into deoxyHb or oxyHb within erythrocytes.

Erythrocytes, whose primary function is oxygen transport, are the most abundant cells in the blood. They lack nuclei, organelles, and cytoplasmic structures, making them unable to synthesize new proteins or proliferate. These cells are the final product of erythropoiesis, a process in which erythrocytes differentiate from hematopoietic stem cells (HSCs) within the bone marrow [8]. Erythrocyte deformability depends on cell geometry (surface/volume ratio), internal viscosity (related to hemoglobin concentration), and the viscoelastic properties of the lipid bilayer and the underlying actin-spectrin network [9,10]. Although erythrocytes lack organized cytoskeletal structures, their membrane-associated actin-spectrin scaffold provides the structural flexibility necessary for navigating capillaries as small as 2–3 μm in diameter [11].

The spectrin network is influenced by shear stress on the erythrocyte membrane, interactions between spectrin and other cytoskeletal proteins, and the availability of chemical energy. Vertical interactions between the lipid bilayer and integral and peripheral proteins—such as band 3, protein 4.2, ankyrin, and the Rh subcomplex—are crucial for maintaining membrane integrity [12,13]. Horizontal interactions that form the spectrin-actin junctional complex involve F-actin-binding proteins like tropomyosin and tropomodulin, as well as specialized proteins such as adducin, protein 4.1 (P4.1), and dematin [14]. Excessive ROS production adversely affects erythrocyte membrane deformability and lipid mobility, leading to protein oxidation and the crosslinking of cytoskeletal and membrane proteins [15].

Additionally, an important factor associated with plasma membrane lipid alterations in patients with essential hypertension is abnormal Na⁺ transport in erythrocytes. This is characterized by increased sodium–lithium exchange, potassium–lithium (K⁺-Li⁺) cotransport, and a higher Li⁺ leak, all of which are significantly elevated in hypertensive individuals [16].

The epithelial sodium channel (ENaC) is a key transporter responsible for sodium reabsorption in the distal nephron, playing a crucial role in the regulation of extracellular fluid volume and, consequently, blood pressure [17]. Its expression in other tissues, including immune cells, has also been found to influence blood pressure through extra-renal mechanisms [18,19].

In a previous study, we identified significant changes in the lipid composition of erythrocyte membranes in patients with hypertension (HTN) compared to normotensive individuals. These changes impacted erythrocyte rigidity, surface roughness, fluidity, and overall biophysical properties. Additionally, we observed elevated oxidative stress in hypertensive erythrocytes [20], as well as in platelets and neutrophils where the ENaC was overexpressed [21,22]. In this study, we identified three distinct erythrocyte subpopulations with differential glycophorin A (GPA) expression. Furthermore, we confirmed the presence of oxidative stress markers in hypertensive erythrocytes and, through Western blotting, cytometry, and confocal microscopy, demonstrated significant alterations in the expression and distribution of key cytoskeletal and integral membrane proteins.

## 2. Materials and Methods

### 2.1. Reagents

Unless otherwise indicated, all reagents were purchased from Sigma Chemical Co. (St. Louis, MO, USA). Table 1 lists the antibodies used in this study.

### 2.2. Study Groups and Ethics Statement

The study population consisted of Blood samples (approximately 10 mL each) from 9 patients with hypertension (HTN) and 9 normotensive individuals (NTI) after obtaining informed consent. The study was approved in advance by the ENMH Ethics Committee (2019–01). and was performed following the Helsinki International ethical standards on human experimentation. All patients enrolled in the study had well-controlled arterial tension values and their blood pressure was monitored weekly by a healthcare professional before and after taking the blood samples. We adopted the blood pressure values of the American College of Cardiology/American Heart Association Hypertension Guideline 2017, therefore, systolic and diastolic values < 120/80 mm Hg were considered normal; elevated blood pressure was defined as 120–129/<80 mm Hg, while HTN was considered as 140/90 mm Hg [23].

After separation of the plasma and buffy coat by centrifugation erythrocytes were isolated from blood samples by centrifugation at 3000 g for 10 min, washed twice with phosphate buffered saline (PBS) and resuspended to the desired hematocrit level in PBS.

Negligible presence of nucleated cells populations was determined, using a gating strategy for analyzing CD11b+ and CD14+ based on forward scatter (FSC-A) and side scatter (SSC-A).

The anthropometric data of the two study groups are presented in Table 2. The hypertensive (HTN) individuals had a mean blood pressure of 145/91 mmHg, while the NTI blood pressure of 115/82 mmHg and no familial history of hypertension. Mean age and body weight (BW) were similar between groups. Exclusion criteria: pregnant women, diabetes, kidney diseases, erythrocytopathies, autoimmune diseases, or hyperlipidemia were not included in this study.

### 2.3. Sample Preparations

Blood samples were drawn by venipuncture in 6 mL tubes (Vacutainer; Becton Dickinson, and Company, Franklin Lakes, NJ, USA) containing EDTA. According to Baskurt et al., EDTA is the most widely used anticoagulant in hemorheological studies [24]. The erythrocytes were isolated by centrifugation from freshly drawn blood at 3000 g for 10 min, and the yellowish supernatant (plasma and white blood cells) was discarded. The remaining erythrocytes were resuspended and washed twice in PBS solution (140 mM NaCl, 2.7 mM KCl, 8 mM Na_2_HPO_4_, 1 mM KH_2_PO_4_), pH 7.4. The hematocrit of the washed erythrocytes suspension was adjusted to 40% in PBS buffer.

### 2.4. Complete Blood Count (CBC)

Automatic cell analyzer, BC6000 (Mindray Medical Mexico, S. DE R. L. DE C; Ciudad de México, Mexico) was used to measure hematological parameters: Red blood cell count (RBC), hemoglobin concentration (Hb), RBC volume percentage (HCT), mean cell volume (MCV), mean cell hemoglobin (MCH), mean corpuscular hemoglobin concentration (MCHC), and absolute as well as percentage number of reticulocytes in all samples.

### 2.5. Quantification of NO Production

Washed HTN and HI erythrocytes (hematocrit, 0.05%) were lysed with distilled water (1:1) and centrifuged (3000 rpm) per 10 min, samples were centrifuged (9000 g) for 1 min, and the concentration of organic nitrites in the supernatant was determined by nitrite colorimetric assay using the Griess reaction at 540 nm [25].

### 2.6. Flow Cytometry Analysis

Erythrocytes from patients with HTN was assessed and compared with that of erythrocytes from NTI were isolated as described above, washed in PBS with 4% fetal bovine serum (FBS), and incubated with primary antibody diluted in PBS with 4% FBS at 4 °C for 30 min followed by a secondary antibody conjugated to Alexa-Fluor-488 (Invitrogen, Waltham, MA, USA), WGA-Rhodamine, and Neutral Red and analyzed by flow cytometry on a LSRFortessa (Beckman Coulter Life Sciences, Indianapolis, IN, USA). Data acquisition and analysis of the erythrocytes subsets was performed using electronic gates to create a templete file that comprised compensation adjustment applied to all the data collected. The first gating strategy used, included a forward and side scatter profiles (plot of FSC-A vs. SSC-A) to identify the erythrocytes population.

Data analysis was performed using Kaluza (Beckman Coulter, Inc., Brea, CA, USA) and mean fluorescence intensity (MFI) values were determined. The Mann–Whitney *U* test was used to compare the significance of the differences.

### 2.7. Immunofluorescence, and Confocal Microscopy

Erythrocytes from patients with HTN and NTI were washed twice with PBS, incubated with methanol at −20 °C for 10 min, and then permeabilized in PBS blocking solution (with 2% bovine serum albumin; Sigma Chemical Co., St. Louis, MO, USA) for 30 min and 0.1% NP40 for 10 min at room temperature. Erythrocytes were incubated for 24 h in a humidified chamber at 4 °C with a primary antibody, washed three times, then incubated at room temperature for 2 h with Alexa 488 or Alexa 568 secondary antibodies diluted in PBS. The primary and secondary antibodies and dilutions are listed in Table 1. After washing, the sections were mounted and observed using a confocal laser scanning microscope LSM 800 (Carl Zeiss, Jena, Germany) with a 40× Plan APO objective (oil immersion 1.3 NA; Zeiss). Negative controls were routinely performed by incubating the samples with only the secondary antibodies to detect nonspecific fluorescence. Sudan black B (0.1% dissolved in 70% ethanol) was used to effectively blocked autofluorescence. Samples from three different individuals (HTN or NTI) were chosen to perform fluorescence relative quantitative analysis using ImageJ, 1.53K.

### 2.8. Ghost Preparation and Western Blotting

Ghosts were prepared from RBC pellets by hypotonic lysis in ~20 volumes ice-cold Mg^2+^-lysis buffer. Membrane skeletons were prepared from ghost pellets by extraction with ~20 volumes of 2% Triton X-100 in ice-cold lysis buffer. Gel samples of ghosts were prepared by the addition of an equal volume of 5× SDS sample buffer and boiling for 5 min [26]. Membrane skeleton pellets were resuspended to the original ghost volume and solubilized as above and subjected to 10% SDS polyacrylamide gel electrophoresis (PAGE), and transferred onto nitrocellulose membranes using a semi-dry system (Thermo Electron Co., Milford, MA, USA). Membranes were exposed to primary antibodies followed by horseradish peroxidase (HRP)-conjugated secondary antibodies and the bands were visualized using an enhanced chemiluminescence Western-blotting analysis system (Santa Cruz Biotechnology, Inc.) and documented using T-mat G/RA film (Kodak, Rochester, NY, USA). Negative controls included transfer strips incubated solely with HRP-conjugated secondary antibodies. Densitometry analysis was performed with Win Image Studio Digits ver. 4.0 software (LI-COR, Inc., Lincoln, NE, USA). Actin was used as a loading control to normalize the data, as its expression showed no significant differences between samples. This was further confirmed by Coomassie staining, which served as an additional normalization method, along with the use of the 95 kDa band from the molecular weight marker BlueEasy Prestained Protein Marker (Cat. No. MWP06, Nippon Genetics Europe, Düren, Germany).

### 2.9. Statistical Analysis

The median fluorescence intensity (MFI) results are expressed as mean ± SEM of nine individuals in each group. Differences between groups were assessed using two-tailed Mann–Whitney for cytometer analysis. For Western-blot and confocal analysis, the results are expressed as mean ± SEM of three individuals in each group. Differences between groups were assessed using the unpaired *t* test, or RM one-way ANOVA (GraphPad Prism 5; GraphPad Software, Inc., La Jolla, CA, USA); differences were considered statistically significant at *p* ≤ 0.05.

## 3. Results

### 3.1. Basic Biometric and Hematological Parameters of HTN Patients and NTI

Table 2 presents the demographic and hematological characteristics of nine patients with hypertension (HTN) and nine normotensive individuals (NTI) used as controls. There were no significant differences in sex or height between the groups. The mean age was 55.6 ± 16.2 years for the HTN group and 47.05 ± 19.3 years for the NTI group. Hematological parameters, including white blood cell (WBC) count, red blood cell (RBC) count, hemoglobin (HGB), hematocrit (HCT), mean corpuscular hemoglobin (MCH), red cell distribution width (RDW), and mean corpuscular hemoglobin concentration (MCHC), were similar between groups. However, the HTN group exhibited a significantly higher mean corpuscular volume (MCV) compared to the NTI group (*p* = 0.0392).

The HTN group had a mean blood pressure of 145/115 mmHg, while the NTI group had a mean blood pressure of 91/82 mmHg, with no personal or familial history of hypertension (Table 2). Patients in the HTN group had been previously diagnosed with essential hypertension and were undergoing treatment. Treatment regimens included angiotensin-converting enzyme (ACE) inhibitors (e.g., enalapril) in four patients (44.4%), angiotensin II receptor antagonists (e.g., losartan, telmisartan) in three patients (33.3%), or a calcium channel blocker (e.g., amlodipine) in two patients (22.2%) (Table 3).

### 3.2. Peroxiredoxin II, a Sensitive Oxidative Stress Marker, Is Dysregulated in Erythrocytes from Patients with Hypertension

**Figure 1:**
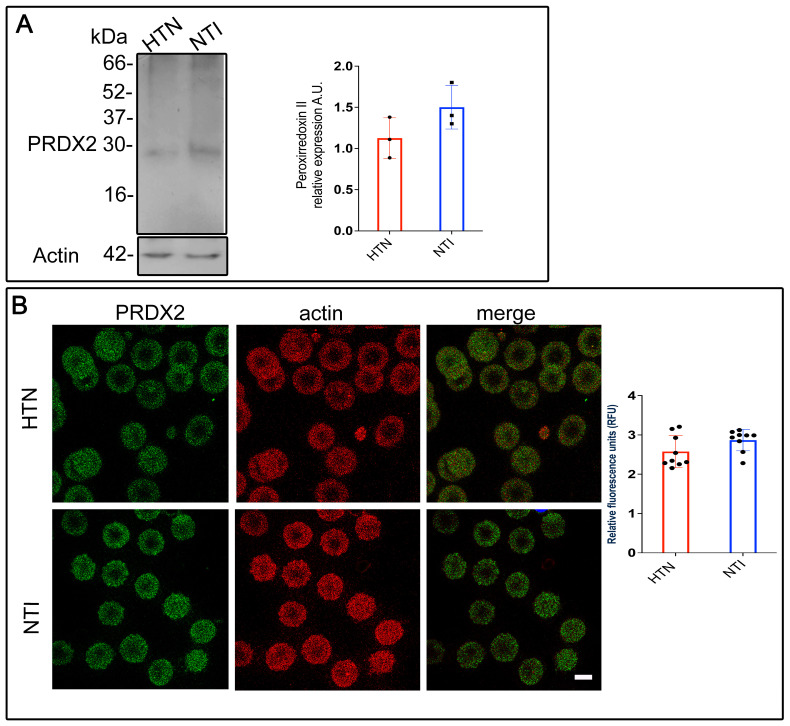
Reduced antioxidant defense mechanisms in erythrocytes from patients with hypertension. (**A**) Membrane skeletons from patients with HTN and NTI were obtained and subjected to Western blot analysis using an anti-peroxiredoxin II antibody (22 kDa). Relative protein expression was quantified using actin as a loading control. Values are presented as mean ± standard error (SE) for three individuals per group. Unpaired *t*-test, *p* = 0.1480. (**B**) Erythrocytes from HTN and NTI were double-labeled with anti-peroxiredoxin II antibody (detected with Alexa-Fluor-488) and anti-actin antibody (detected with Alexa-Fluor 568). Seventy-five cells from three independent experiments were analyzed by confocal microscopy. Scale bar = 5 µm. Relative fluorescence units demonstrated a down-expression of PRDX2 in erythrocytes from HTN patients. Values shown are mean ± Standard Error (SE). Unpaired *t* test.

The oxygen-transporting function of hemoglobin within erythrocytes is highly vulnerable to oxidative [27]. However, erythrocytes are equipped with peroxiredoxin II (PRDX2), an antioxidant enzyme that neutralizes reactive oxygen species (ROS) generated internally and in tissues they interact with. To investigate whether PRDX2 is sufficient to counteract ROS in erythrocytes from hypertensive (HTN) patients, membrane skeletons from erythrocytes of HTN patients and normotensive individuals (NTI) were analyzed via Western blotting. A ~22 kDa band corresponding to PRDX2 was detected in both groups. However, the band intensity in erythrocyte membrane skeletons from HTN patients was lower compared to NTI ($\bar{x}$ = 1.13 ± 0.143 vs. 1.50 ± 0.153; *p* = 0.1480; Figure 1A).

To further examine PRDX2 distribution, double immunofluorescent staining was performed on erythrocytes from HTN patients and NTI. An anti-PRDX2 antibody labeled with Alexa Fluor 488 and an anti-actin antibody labeled with Alexa Fluor 568 were used. Confocal microscopy revealed a fine, patchy cytoplasmic distribution of PRDX2 in erythrocytes from both groups, with no notable differences in actin distribution (Figure 1B). Merged images showed no co-localization of PRDX2 with actin in either HTN or NTI erythrocytes. Relative quantification using confocal microscopy confirmed a downward trend in PRDX2 expression in erythrocytes from HTN patients compared to NTI ($\bar{x}$ = 2.576 ± 0.1366 vs. 2.867 ± 0.09061; *p* = 0.0948; Figure 1B).

In summary, these findings demonstrate that PRDX2 expression is reduced in erythrocytes from HTN patients, suggesting a dysregulated oxidative stress response.

### 3.3. 3-Nitrotyrosine (3-NT), a Specific Marker of Oxidative Protein Damage, Is Overexpressed in Erythrocytes from Hypertensive Patients

**Figure 2:**
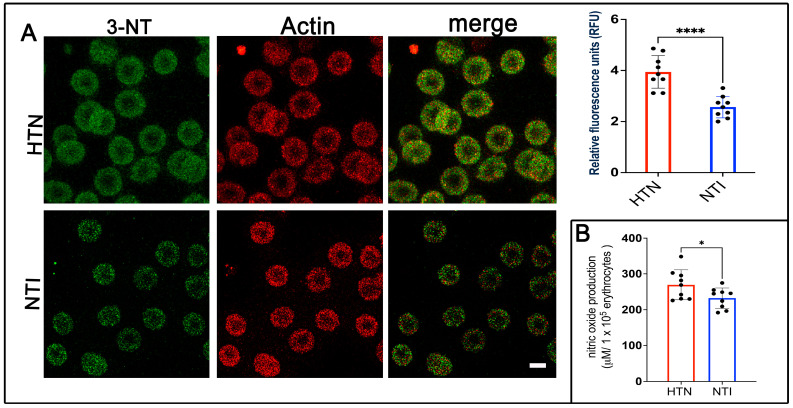
Overexpression of 3-nitrotyrosine (3-NT) in erythrocytes from patients with hypertension. (**A**) Erythrocytes from HTN and NTI were analyzed by confocal microscopy after immunofluorescence labeling with anti-3-NT (detected with Alexa-Fluor-488) and anti-actin (detected with Alexa-Fluor 568). Scale bar = 5 µm. Relative fluorescence units (RFU) showed a significant upregulation of 3-NT in 75 erythrocytes from three observation fields of HTN compared to NTI. Data are expressed as mean ± SE from three independent experiments (n = 3). Unpaired *t*-test, **** *p* < 0.0001. (**B**) Nitric oxide quantification using the Griess reaction revealed higher levels in erythrocytes from HTN compared to NTI. Unpaired *t*-test, * *p* = 0.0421.

**Figure 3:**
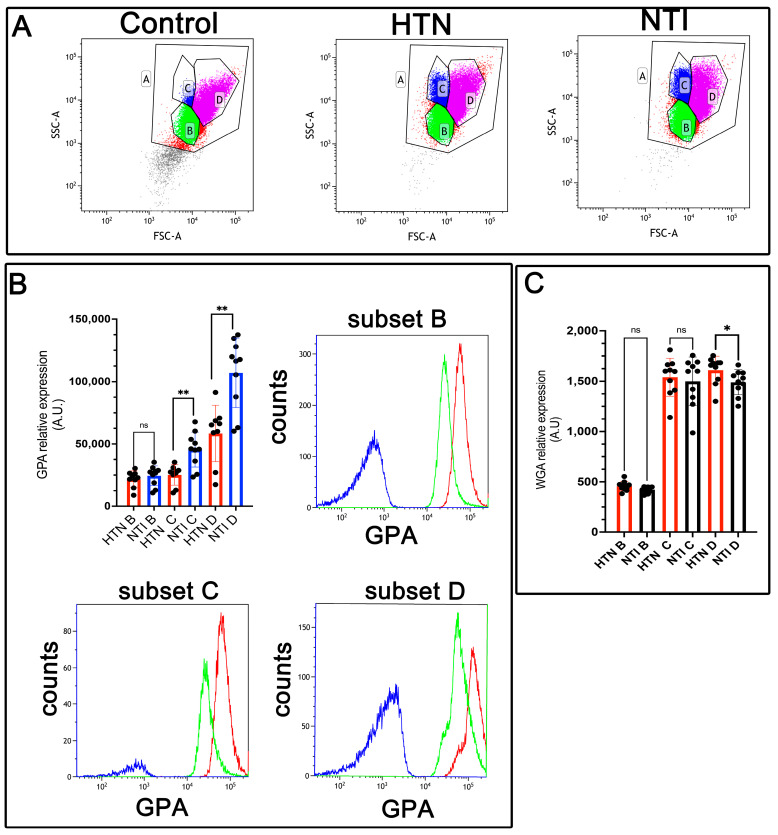
Decreased expression of glycophorin A (GPA) in erythrocytes from hypertensive patients. (**A**). Identification of three erythrocyte subsets based on forward and side scatter profiles (FSC-A vs. SSC-A) showing the whole population (A) and subsets (B, C, D) in control erythrocytes (without GPA antibody), and in GPA-labeled erythrocytes from HTN and NTI. (**B**). Relative quantification of GPA in erythrocyte subsets (B, C, D) from HTN and NTI. *p* = 0.2428T; ** *p* = 0.0076; ** *p* = 0.0041. Histograms for each subset are depicted. (**C**). WGA staining in erythrocyte subsets (B, C, D) from HTN and NTI. *p* = 0.1181; *p* = 0.9999; * *p* = 0.0037.

Nitration of protein tyrosine residues, resulting in the formation of 3-nitrotyrosine (3-NT), can alter protein function, leading to either gain or loss of activity [28]. It is widely recognized as a biomarker of reactive nitrogen species, peroxynitrite formation, and nitric oxide (NO) production. To assess 3-NT expression, erythrocytes from hypertensive (HTN) patients and normotensive individuals (NTI) were subjected to double immunofluorescence labeling. Antibodies against 3-NT (detected with Alexa Fluor 488) and actin (detected with Alexa Fluor 568) were used. Confocal microscopy revealed a fine punctate distribution of 3-NT in the cytoplasm and plasma membrane of erythrocytes from HTN patients, which was also present in erythrocytes from NTI (Figure 2A). Quantification of fluorescence from 75 erythrocytes per group across three independent experiments demonstrated significantly elevated 3-NT expression in erythrocytes from HTN patients compared to NTI ($\bar{x}$ = 3.905 ± 0.3863 vs. $\bar{x}$ = 2.811 ± 0.1659; *p* = 0.0315; Figure 3A). Actin distribution remained consistent between the two groups, and merged images revealed no apparent colocalization between 3-NT and actin (Figure 2A).

To evaluate NO levels, the Griess reaction was performed, which chemically reduces nitrate to nitrite for detection [25]. Serial dilutions from 50 to 400 µM showed a linear correlation between absorbance and nitrite concentration. Results indicated significantly higher NO levels in erythrocytes from HTN patients compared to NTI ($\bar{x}$ = 269.5 ± 13.91 vs. $\bar{x}$ = 232.4 ± 9.44; * *p* = 0.0421; Figure 2B).

These findings highlight increased 3-NT and NO levels in erythrocytes from HTN patients, providing important insights into the oxidative stress and cellular damage associated with hypertension pathogenesis.

### 3.4. Low Expression of Glycophorin A (GPA) and Sialic Acid Content Is Associated with Increased Erythrocyte Aggregation in Hypertensive Patients

Glycophorin A (GPA), a sialoglycoprotein abundant in the erythrocyte membrane, is rich in sialic acid, which contributes to the cell’s net negative surface charge. This charge minimizes erythrocyte–erythrocyte interactions, thereby preventing [29]. However, erythrocyte aggregation is enhanced in hypertension [30].

To evaluate GPA expression, erythrocytes from hypertensive (HTN) patients and normotensive individuals (NTI) were analyzed by flow cytometry using an anti-GPA antibody and an Alexa Fluor 488-conjugated secondary antibody. Based on forward scatter (FSC) measurements, three distinct erythrocyte populations (B, C, and D) were identified (Figure 3A). Median fluorescence intensity (MFI) for GPA in population B from HTN erythrocytes showed a slight reduction compared to NTI, although the difference was not statistically significant ($\bar{x}$ = 21,867 ± 2178 vs. $\bar{x}$ = 24,484 ± 2493; *p* = 0.2428). However, GPA expression was significantly reduced in subpopulations C and D of HTN erythrocytes compared to NTI ($\bar{x}$ = 25,116 ± 2697 vs. $\bar{x}$ = 45,464 ± 4436; *p* = 0.0076, and $\bar{x}$ = 58,403 ± 7558 vs. $\bar{x}$ = 107,057 ± 8807; * *p* = 0.0041, respectively) (Figure 3B).

To investigate changes in sialoglycoproteins, we used wheat germ agglutinin (WGA), a lectin with high specificity for N-acetylglucosamine and N-acetylneuraminic acid (sialic acid) residues [31]. Erythrocytes from HTN patients and NTI were labeled with fluorescent WGA and analyzed by flow cytometry. As with GPA, three distinct erythrocyte subpopulations were identified. Subpopulations B and C from HTN erythrocytes showed similar but slightly lower WGA MFI values compared to NTI ($\bar{x}$ = 457.5 ± 15.04 vs. $\bar{x}$ = 422.8 ± 10.11; * *p* = 0.1181, and $\bar{x}$ = 1499 ± 76.34 vs. $\bar{x}$ = 1499 ± 76.34; * *p* = 0.9999, respectively). Notably, subpopulation D from HTN patients exhibited a significantly higher WGA MFI compared to NTI ($\bar{x}$ = 1611 ± 43.68 vs. $\bar{x}$ = 1490 ± 39.43; * *p* = 0.0371) (Figure 3C).

These results suggest that diminished sialic acid levels in erythrocytes from HTN patients may be counterbalanced by an increased expression of sialoglycoproteins. This compensatory mechanism might help mitigate the enhanced erythrocyte aggregation observed in hypertension

### 3.5. Oxidative Damage to Spectrin Alters Its Subcellular Distribution in Hypertensive Patients

Previous findings have demonstrated that erythrocytes from hypertensive (HTN) patients experience higher oxidative stress compared to those from normotensive individuals (NTI) [20].

Oxidative damage to cytoskeletal proteins, such as spectrin, has been implicated in altered erythrocyte shape and reduced membrane elasticity. Spectrin, a key component of the erythrocyte membrane skeleton, interacts with both peripheral and integral membrane proteins to maintain structural integrity.

**Figure 4:**
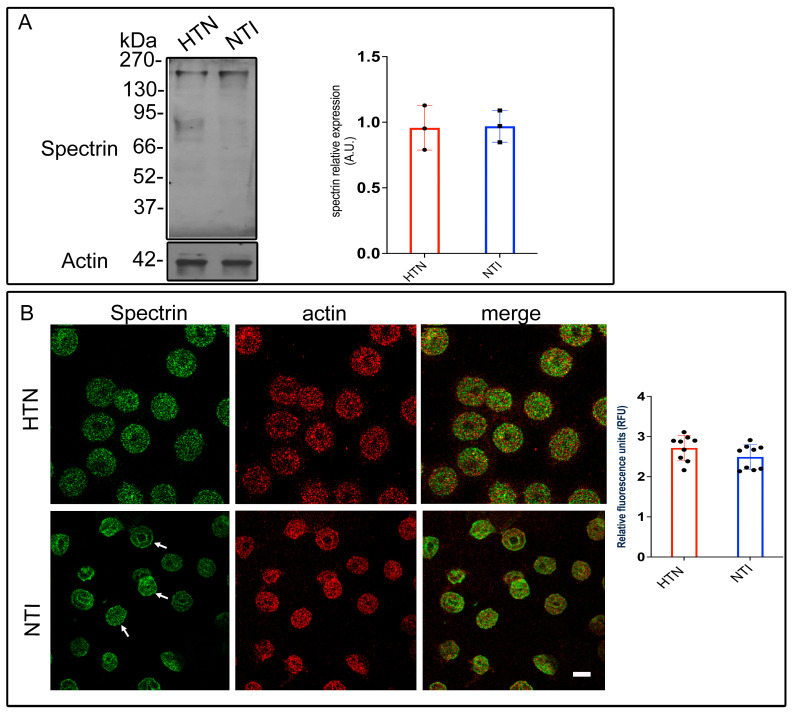
Altered subcellular distribution of spectrin in erythrocytes from hypertensive patients. (**A**) Erythrocytes from HTN and NTI were lysed and processed for Western blot analysis using an anti-spectrin antibody (230 kDa). Relative protein expression was quantified using actin as a loading control. Values are presented as mean ± SE for three individuals per group. Unpaired *t*-test, *p* = 0.9236. (**B**) Confocal microscopy analysis of erythrocytes from HTN and NTI double-labeled with anti-spectrin (Alexa-Fluor-488) and anti-actin (Alexa-Fluor 568). Seventy-five cells from three observations field and three independent experiments were analyzed. Scale bar = 5 µm. Values shown are mean ± Standard Error (SE). Unpaired *t* test, *p* = 0.1441. White arrows show the homogeneous cytoplasmic distribution of spectrin at the plasma membrane.

To investigate spectrin modifications in HTN patients, erythrocyte membrane skeletons from five HTN patients and five NTI were analyzed by Western blotting using an anti-spectrin antibody. Bands corresponding to spectrin (~230 kDa) displayed similar intensities in both groups. Densitometric analysis revealed no significant differences in band intensity between HTN and NTI erythrocytes ($\bar{x}$ = 0.957 ± 0.0976 vs. $\bar{x}$ = 0.969 ± 0.0699; *p* = 0.9236; Figure 4A).

To assess spectrin distribution, erythrocytes from HTN and NTI groups were double-labeled with an anti-spectrin antibody conjugated to Alexa Fluor 488 and an anti-actin antibody conjugated to Alexa Fluor 568. Confocal microscopy revealed distinct patterns of spectrin distribution. In NTI erythrocytes, spectrin displayed a homogeneous cytoplasmic distribution with clear localization to patches at the plasma membrane (indicated by arrows) and around the biconcave regions. In HTN erythrocytes, spectrin was observed in a fine punctate cytoplasmic pattern. Actin exhibited a patched distribution in both HTN and NTI samples, with similar levels of co-localization with spectrin in both groups (Figure 4B).

Quantitative confocal microscopy analysis indicated a trend toward increased spectrin levels in erythrocytes from HTN patients compared to NTI, although this difference was not statistically significant ($\bar{x}$ = 2.718 ± 0.1049 vs. 2.493 ± 0.1016; * *p* = 0.1441; Figure 4B).

These findings suggest that oxidative stress does not quantitatively affect spectrin expression in HTN erythrocytes, as determined by gel electrophoresis. However, confocal microscopy highlights potential qualitative alterations in its subcellular distribution, which may reflect oxidative damage affecting spectrin’s interaction with other cytoskeletal components or the erythrocyte membrane.

### 3.6. Reduced Anion Exchange Capacity of Band 3 Protein in Erythrocytes from Hypertensive Patients

Band 3 protein (B3p), also known as anion exchanger 1 (AE1), is a crucial erythrocyte membrane protein responsible for chloride-bicarbonate exchange, maintaining ionic balance, and supporting erythrocyte deformability. This deformability is essential for efficient tissue oxygenation and has been linked to cardiovascular health [32]. Given the potential role of erythrocyte deformability in hypertension, we evaluated B3p expression and distribution in erythrocytes from hypertensive (HTN) patients compared to normotensive individuals (NTI).

**Figure 5:**
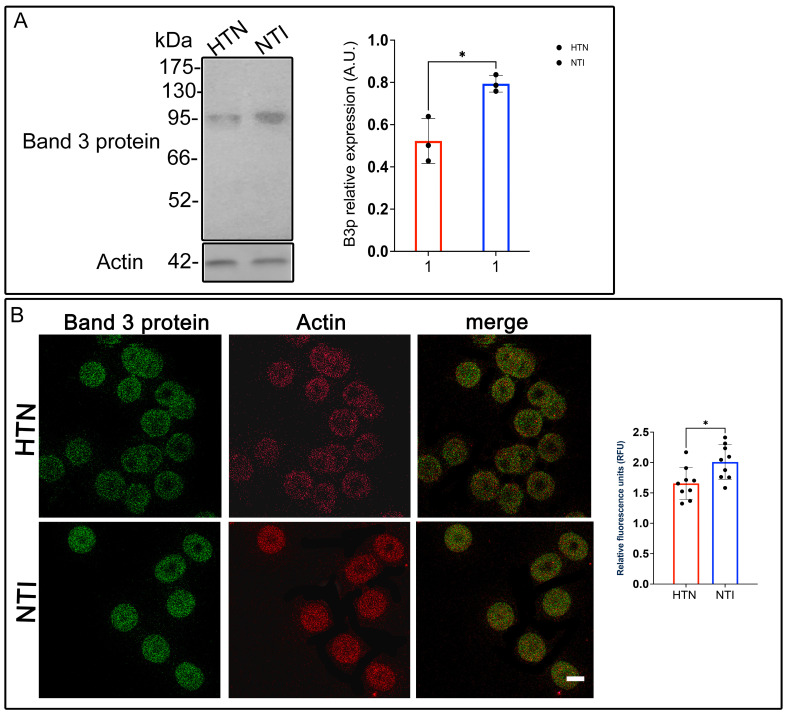
Reduced expression of Band 3 protein in erythrocytes from hypertensive patients. (**A**) Erythrocytes from HTN and NTI were lysed and processed for Western blot analysis using an anti-Band 3 protein antibody (93 kDa). Relative protein expression was quantified using actin as a loading control. Values are presented as mean ± SE for three individuals per group. Unpaired *t*-test, * *p* = 0.0146. (**B**) Confocal microscopy analysis of erythrocytes from HTN and NTI double-labeled with anti-Band 3 (Alexa-Fluor-488) and anti-actin (Alexa-Fluor 568). Seventy-five cells from three independent experiments and three observational fields were analyzed. Scale bar = 5 µm. Values shown are mean ± Standard Error (SE). Unpaired *t* test, * *p* = 0.0147.

Western blot analysis of erythrocyte membrane skeletons identified a ~93 kDa band corresponding to B3p. Quantitative densitometry revealed a significant reduction in B3p levels in erythrocytes from HTN patients relative to NTI ($\bar{x}$ = 0.5229 ± 0.0616 vs. $\bar{x}$ = 0.7838 ± 0.228; * *p* = 0.0146; Figure 5A).

To investigate B3p localization and its relationship with actin, double immunofluorescent staining was performed using anti-B3p and anti-actin antibodies conjugated to Alexa Fluor dyes. Confocal microscopy showed no discernible differences in the distribution patterns of B3p between HTN and NTI erythrocytes. Actin exhibited a consistent localization in both groups, with minimal co-localization observed between actin and B3p (Figure 5B).

Interestingly, relative quantification via confocal microscopy showed a significant overexpression of B3p in erythrocytes from HTN patients compared to NTI ($\bar{x}$ = 1.657 ± 0.0879 vs. $\bar{x}$ = 2.009 ± 0.09427; * *p* = 0.0147; Figure 5B).

These results highlight a complex scenario where B3p abundance, as measured by Western blotting, is reduced in HTN erythrocytes, while its fluorescence intensity suggests potential changes in protein conformation, accessibility, or post-translational modifications. Such alterations in B3p may compromise its anion exchange function, contributing to reduced erythrocyte deformability and oxygen transport efficiency in hypertensive patients.

### 3.7. Protein 4.1R, a Key Connector in the Erythrocyte Membrane Skeleton, Is Overexpressed in Hypertensive Patients

**Figure 6:**
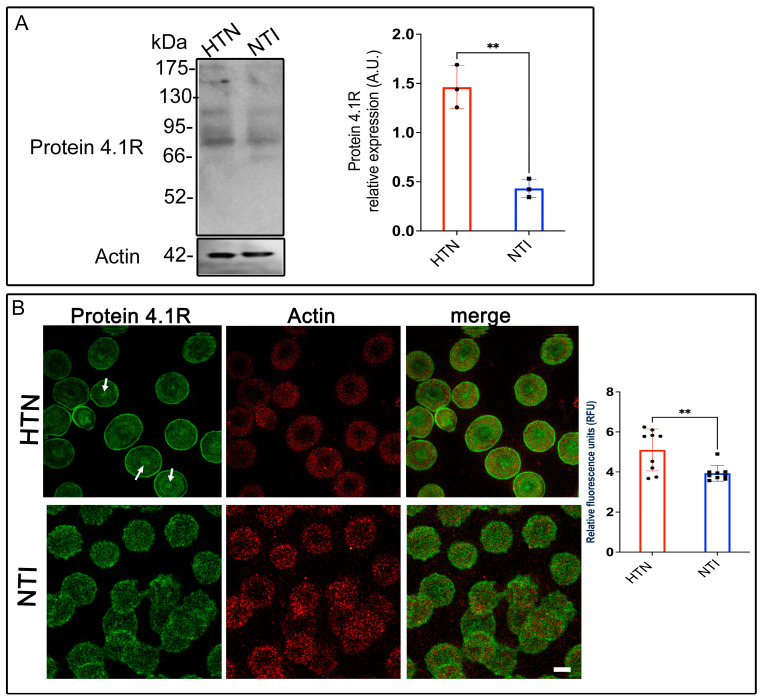
Increased expression of protein 4.1R in erythrocytes from hypertensive patients. (**A**). Erythrocytes from HTN and NTI were lysed and processed for Western blot analysis using an anti-protein 4.1R antibody (80 kDa). Relative protein expression was quantified using actin as a loading control. Values are presented as mean ± SE for three individuals per group. Unpaired *t*-test, ** *p* = 0.017. (**B**). Confocal microscopy analysis of erythrocytes from HTN and NTI double-labeled with anti-protein 4.1R (Alexa-Fluor-488) and anti-actin (Alexa-Fluor 568). Seventy-five cells from three independent experiments were analyzed. Scale bar = 5 µm. samples. Values shown are mean ± Standard Error (SE). Unpaired *t* test, ** *p* = 0.0065.

Erythrocyte protein 4.1R (4.1R) is a critical cytoskeletal component that ensures cell stability and flexibility by anchoring spectrin, F-actin, and Band 3 protein (B3p) to the plasma membrane [33]. Given the alterations in other cytoskeletal and membrane proteins observed in erythrocytes from hypertensive (HTN) patients, we evaluated 4.1R expression in this population. Western blot analysis revealed a band at ~80 kDa corresponding to 4.1R in erythrocytes from HTN patients and normotensive individuals (NTI). Densitometric quantification indicated a significant overexpression of 4.1R in HTN erythrocytes compared to NTI ($\bar{x}$ = 1.462 ± 0.1254 vs. $\bar{x}$ = 0.4324 ± 0.05428; *p* = 0.017; Figure 6A). Confocal microscopy revealed distinct distribution patterns of 4.1R in HTN and NTI erythrocytes. In HTN erythrocytes, 4.1R exhibited a fine punctate pattern concentrated at the plasma membrane and discrete central accumulations (Figure 6B, arrows). In contrast, NTI erythrocytes displayed a coarser punctate pattern that was more uniformly distributed throughout the cytoplasm and plasma membrane (Figure 6B). Despite these differences, actin distribution remained consistent across both groups, with no apparent co-localization observed between actin and 4.1R. Quantitative analysis of confocal microscopy data confirmed a significant overexpression of 4.1R in HTN erythrocytes compared to NTI ($\bar{x}$ = 5.098 ± 0.3493 vs. $\bar{x}$ = 3.932 ± 0.1308; * *p* = 0.0065; Figure 6B).

These findings suggest that protein 4.1R might play a compensatory role in HTN erythrocytes, potentially counteracting the damage and structural instability caused by oxidative stress to other cytoskeletal components. Enhanced expression of 4.1R may reflect an adaptive mechanism to maintain membrane integrity and erythrocyte deformability under hypertensive conditions.

### 3.8. Ankyrin ExpressionEexhibits a Tendency to Increase in Erythrocytes from Patients with Hypertension

Ankyrin, a critical adaptor protein, connects spectrin to Band 3 protein (B3p), anchoring the cytoskeleton to the erythrocyte membrane through transmembrane proteins [34]. Given the changes observed in spectrin and B3p in erythrocytes from hypertensive (HTN) patients, we investigated whether ankyrin expression and distribution were similarly affected.

**Figure 7:**
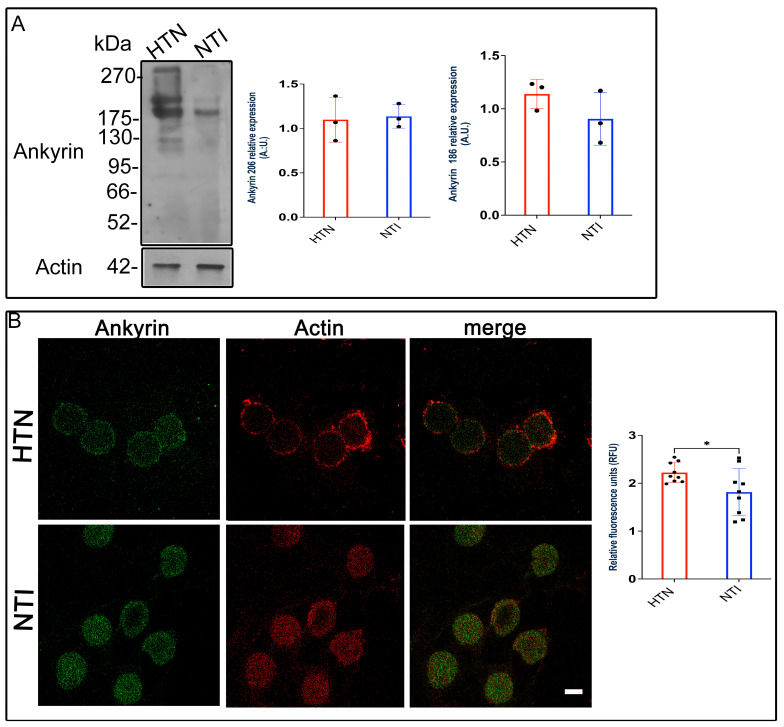
Ankyrin levels remain unchanged in erythrocytes from hypertensive patients. (**A**). Erythrocytes from HTN and NTI were lysed and processed for Western blot analysis using an anti-ankyrin antibody (186 kDa and 206 kDa). Relative protein expression was quantified using actin as a loading control. Values are presented as mean ± SE for three individuals per group. Unpaired *t*-test, *p* = 0.2235 (186 kDa) and *p* = 0.8319 (206 kDa). (**B**). Confocal microscopy analysis of erythrocytes from HTN and NTI double-labeled with anti-ankyrin (Alexa-Fluor-488) and anti-actin (Alexa-Fluor 568). Seventy-five cells from three observation fields and three independent experiments were analyzed. Scale bar = 5 µm. Values shown are mean ± Standard Error (SE). Unpaired *t* test, * *p* = 0.0336.

Western blot analysis identified ankyrin bands at approximately ~186 kDa and ~206 kDa. Quantitative densitometry revealed no significant differences in ankyrin expression between HTN and normotensive individuals (NTI) for either the 186 kDa band ($\bar{x}$ = 0.9051 ± 0.1424 vs. $\bar{x}$ = 1.139 ± 0.07899; *p* = 0.2235) or the 206 kDa band ($\bar{x}$ = 1.098 ± 0.1459 vs. $\bar{x}$ = 1.135 ± 0.07646; *p* = 0.8319; Figure 7A).

To assess ankyrin distribution, we performed double immunofluorescence staining using Alexa-Fluor 488-labeled anti-ankyrin and Alexa-Fluor 568-labeled anti-actin antibodies. Confocal microscopy revealed similar ankyrin distribution patterns in HTN and NTI erythrocytes, with no co-localization observed between ankyrin and actin (Figure 7B).

Interestingly, relative quantification of fluorescence intensity by confocal microscopy indicated a statistically significant increase in ankyrin expression in HTN erythrocytes compared to NTI ($\bar{x}$ = 1.766 ± 0.1837 vs. $\bar{x}$ = 2.222 ± 0.0689; *p* = 0.0366; Figure 7B).

These findings suggest that while Western blot analysis does not indicate major alterations in ankyrin protein levels, confocal microscopy data reveal a modest increase in ankyrin expression in HTN erythrocytes. This increase may reflect subtle compensatory mechanisms within the erythrocyte cytoskeleton under hypertensive conditions.

### 3.9. ENaC Is Overexpressed in Erythrocytes from Patients with Hypertension (HTN)

**Figure 8:**
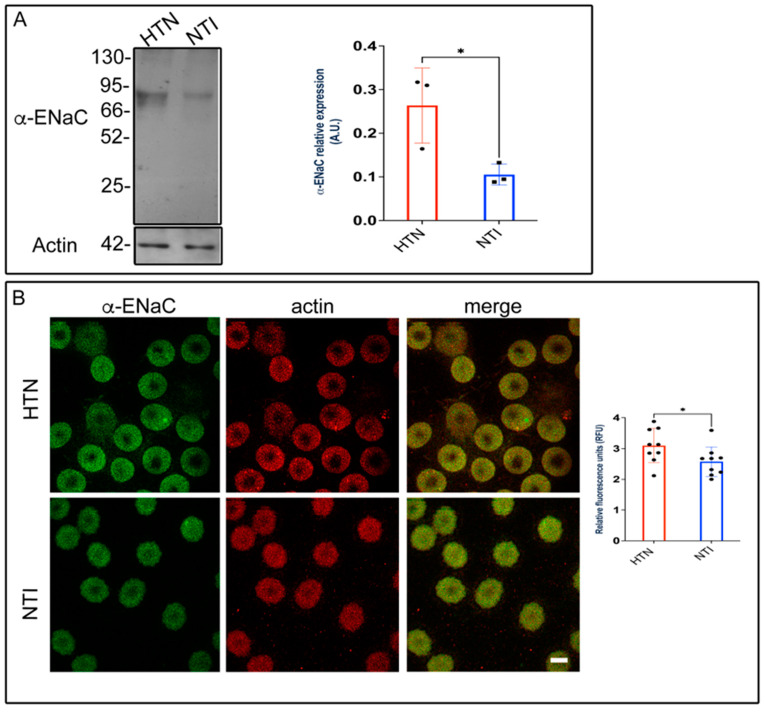
Overexpression of ENaC in erythrocytes from hypertensive patients. (**A**) Erythrocytes from HTN and NTI were lysed and processed for Western blot analysis using an anti-ENaC antibody (75 kDa). Relative protein expression was quantified using actin as a loading control. Values are presented as mean ± SE for three individuals per group. Unpaired *t*-test, * *p* = 0.0373. (**B**) Confocal microscopy analysis of erythrocytes from HTN and NTI double-labeled with anti-ENaC (Alexa-Fluor-488) and anti-actin (Alexa-Fluor 568). Seventy-five cells from three observational fields and three independent experiments were analyzed. Scale bar = 5 µm. Values shown are mean ± Standard Error (SE). Unpaired *t* test, * *p* = 0.0491.

Alterations in membrane composition can significantly impact membrane permeability, transport systems, receptor functions, and enzyme activities [35,36]. Previously, we demonstrated that plasma membrane lipid composition and fluidity in red blood cells (RBCs) were reduced in patients with hypertension, potentially contributing to the pathophysiology of this condition [20]. To investigate the involvement of the epithelial sodium channel (ENaC), we assessed the expression of its α-subunit using Western blotting and immunofluorescence. Western blot analysis identified a band at approximately ~75 kDa, which was significantly overexpressed in erythrocytes from hypertensive (HTN) patients compared to normotensive individuals (NTI) ($\bar{x}$ = 0.264 ± 0.0497 vs. $\bar{x}$ = 0.105 ± 0.138; *p* = 0.0373; Figure 8A). Immunofluorescence analysis revealed that ENaC was distributed in a fine, homogeneous patch-like pattern within the cytoplasm of erythrocytes from both hypertensive (HTN) patients and normotensive individuals (NTI). Actin distribution remained unaffected, with no observable differences between the two groups (Figure 8B). In merged images, ENaC patches were discretely and homogeneously distributed within the cytoplasm. Relative quantification by confocal microscopy demonstrated a significant overexpression of α-ENaC in erythrocytes from HTN patients compared to NTI ($\bar{x}$ = 3.098 ± 0.1855 vs. $\bar{x}$ = 2.576 ± 0.1601; *p* = 0.0491; Figure 8B).

These findings suggest that ENaC may contribute to elevated intracellular Na+ levels in erythrocytes from patients with HTN.

## 4. Discussion

Hypertension (HTN) is a major cardiovascular risk factor [37], and reactive oxygen species (ROS) play a key role in both the regulation and progression of cardiovascular diseases. Oxidative stress has been identified as a significant contributor to the development and progression of this chronic condition [38]. Erythrocytes, primarily responsible for gas exchange, are continually exposed to high oxygen tension, making them susceptible to oxidative damage. Previous studies have shown that erythrocytes from patients with HTN exhibit elevated oxidative stress, characterized by increased ROS and decreased glutathione (GSH) levels, compared to those from normotensive individuals (NTI) [20]. In this study, we observed a trend toward decreased PRDX2 levels in erythrocytes from HTN patients. This reduction may be linked to increased 3-nitrotyrosine (3-NT) levels, elevated nitric oxide (NO) production, and heightened oxidative stress—all factors implicated in the pathophysiology of HTN. Circulating erythrocytes, which lack organelles, are unable to repair irreversible lipid and protein damage, leading to compromised functionality and eventual membrane rupture or hemolysis [20,39]. Nitric oxide (NO), synthesized in both erythrocytes and endothelial cells [40], appears to act as a compensatory mechanism in hypertensive conditions, counteracting oxidative stress and mitigating hemolysis and endothelial dysfunction [41]. Increased NO synthesis in erythrocytes from HTN patients may enhance membrane fluidity and reduce rigidity compared to NTI. However, the significant alterations observed in hypertension—such as reduced erythrocyte deformability, increased aggregation, and decreased oxygen transport to tissues—may exacerbate the condition. These changes could also contribute to the observed increase in erythrocyte size.

Mean corpuscular volume (MCV) reflects the average volume of an erythrocyte, while red cell distribution width (RDW) represents the standard deviation of MCV. Both parameters have been proposed as potential predictors for cardiovascular and cerebrovascular diseases [42,43]. In this study, patients with hypertension (HTN) exhibited no significant changes in RDW but showed an increased MCV, which was the only altered parameter when compared to normotensive individuals (NTI). This finding is consistent with observations of low or normal hematocrit, a key determinant of whole blood viscosity [44]. Elevated MCV in erythrocytes may also indicate the presence of younger cells, as aging erythrocytes undergo a progressive loss of surface area and volume [45].

The reduction in glycophorin A (GPA) expression across all three erythrocyte subpopulations in patients with hypertension suggests an increased tendency for aggregation [46]. Notably, the largest subpopulation, which exhibits a significant decrease in GPA expression, poses a heightened risk for erythrocytes to aggregate more readily. These findings highlight the need for clinical attention to prevent potential complications in hypertensive patients. This increased aggregation could compromise blood flow and oxygen transport [47], exacerbating the vascular dysfunction associated with hypertension. Moreover, alterations in the typical biconcave shape of erythrocytes from HTN patients, along with reduced cell volume, impair gas exchange and deformability compared to NTI [48]. We propose that the elevated expression of N-acetylglucosamine and N-acetylneuraminic acid, detected by cytometry with wheat germ agglutinin (WGA), could be a compensatory mechanism aimed at counteracting the heightened erythrocyte aggregability in HTN patients, which may further hinder blood flow and oxygen delivery. Additionally, the lower cholesterol content in the plasma membranes of erythrocytes from HTN patients [20] suggests a disruption in the association between GPA and band 3 protein (B3p), as cholesterol typically reduces the lateral mobility of lipids and proteins within the membrane [49].

Additionally, the lower cholesterol content in the plasma membranes of erythrocytes from HTN patients, in comparison to normotensive individuals (NTI). Since band 3 protein (B3p) plays a critical role in the osmotic and mechanical properties of erythrocytes [50], its reduction may disrupt the alignment between the lipid bilayer and the cytoskeleton, ultimately leading to a loss of the membrane’s typical structure, as observed in our confocal analysis when compared to erythrocytes from normotensive individuals (NTI). On the other hand, the reduction in B3p may trigger the overexpression of protein 4.1, which stabilizes interactions between the plasma membrane and the membrane skeleton, as reported by Salomao et al. (2008) [51]. This phenomenon was primarily observed at the plasma membrane in our study.

Decreased red blood cell (RBC) deformability in hypertensive individuals has been linked to impaired sodium ion regulation [52]. Elevated sodium concentrations have been shown to correlate with increased blood pressure levels, likely due to reduced Na⁺-pump activity [53]. Based on our results, it is plausible that the increased intracellular sodium in HTN patients is a consequence of the overexpression of the α-subunit of the epithelial sodium channel (α-ENaC), as has been observed in other blood cell lineages from hypertensive individuals [21,22].

Our study reveals alterations in key proteins, such as spectrin, glycophorin A, band 3, and band 4.1, which have been implicated in hereditary anemia. We acknowledge that an important limitation of our study is the small number of patients included. Nevertheless, our findings demonstrate modifications in the cytoskeleton of erythrocytes from patients with hypertension (HTN), which can be attributed to oxidative stress, as previously described during the storage of erythrocytes for transfusion [54]. These changes include thicker spectrin filaments and a loss of connections [55]. Therefore, future investigations should focus on studying alterations in the interactions between band 3 and spectrin, band 3 and ankyrin, and spectrin and protein 4.1.

Furthermore, additional research is needed to explore how these cytoskeletal changes affect the viscoelastic properties of the cell membrane and their relationship to vascular dysfunction in hypertension. Given the role of oxidative stress in erythrocyte dysfunction, antioxidant interventions may hold therapeutic potential for reversing these cytoskeletal alterations and improving erythrocyte function in hypertensive patients.

## 5. Conclusions

Our study provides novel insights into the cytoskeletal and membrane protein alterations in erythrocytes from hypertensive patients. We identified three distinct erythrocyte subpopulations with differential glycophorin A expression, which, in conjunction with oxidative stress markers, revealed significant disruptions in the expression and distribution of key proteins such as spectrin, Band 3, protein 4.1R, and ankyrin. These alterations suggest that oxidative stress plays a pivotal role in modulating the structural integrity of the erythrocyte membrane, potentially impairing its viscoelastic properties and overall function. This compromised membrane elasticity could contribute to the vascular dysfunction observed in hypertension. Our findings underscore the need for further investigation into the molecular mechanisms underlying these alterations and suggest that therapeutic strategies targeting oxidative stress may offer potential for restoring erythrocyte function in hypertensive patients.

## Figures and Tables

**Table 1:** Characteristics of antibodies used.

Antibody	Catalog Num	Specificity	Dilution Wb/IF/IP	Resource
anti-α-ENaC	PA1-920A	P	WB 1:100 IF 1:50	Invitrogen (Waltham, MA, USA)
anti-Actin	SC-8432	M	WB 1:100 IF 1:50	Santa Cruz Biotechnology (Santa Cruz, CA, USA)
anti-4.1R	SC-166759	M l	WB 1:100 IF 1:50	Santa Cruz Biotechnology (Santa Cruz, CA, USA)
anti-Band 3	SC-133190	M	WB 1:100 IF 1:50	Santa Cruz Biotechnology (Santa Cruz, CA, USA)
anti-Spectrin	SC-53444	M	WB 1:100 IF 1/50	Santa Cruz Biotechnology (Santa Cruz, CA, USA)
anti-Ankyrin	SC-374105	M	WB 1:100 IF 1:50	Santa Cruz Biotechnology (Santa Cruz, CA, USA)
anti-PRDX2	SC-515428	M	WB 1:100 IF 1:50	Santa Cruz Biotechnology (Santa Cruz, CA, USA)
Anti Glycophorin A	SC-53295	M	Cytometry 1:100	Santa Cruz Biotechnology (Santa Cruz, CA, USA)
Anti-nytrotyrosine	ab110282	M	IF: 1:80	Abcam (Waltham, MA, USA)
CD11 b PE	cat No. 557397	M	Cytometry 1:100	BD Biosciences (San Jose, CA, USA)
CD45	cat No. 555480	M	Cytometry 1:100	BD Biosciences (San Jose, CA, USA)
Anti-mouse alexa488	A10684	--	IF 1:150	Invitrogen (Waltham, MA, USA)
Anti-rabbit alexa568	A10042	--	IF 1:150	Invitrogen (Waltham, MA, USA)
Goat Anti-Mouse IgG-HRP	115-035-003	--	WB 1:4000	Jackson ImmunoResearch Laboratories (West Grove, PA, USA)
Goat Anti-Rabbit IgG-HRP	111-035-003	--	WB 1:4000	Jackson ImmunoResearch Laboratories (West Grove, PA, USA)

M: monoclonal; P: polyclonal.

**Table 2:** Patient demographics and laboratory values.

Parameter	HTN	NTI	*p* Value
Males/Females (n)	4/5	5/4	-----
Age	55.6 ± 16.2	47.05 ± 19.3	0.1792
SBP (mm/Hg)	145 ± 20	115 ± 5	**0.0015**
DBP (mm/Hg)	91 ± 10	82 ± 4	**0.001**
WBC (×10^3^/μL)	6.95 ± 1.54	7.97 ± 2.19	0.1805
RBC (×10^6^/μL)	4.43 ± 0.67	4.86 ± 0.49	0.0853
HGB (g/dL)	14.01 ± 2.01	14.64 ± 1.63	0.4525
HCT (%)	41.61 ± 6.29	42.8 ± 4.06	0.2695
MCV (fL)	92.55 ± 3.54	88.98 ± 5.53	**0.0392**
MCHC (g/dL)	32.37 ± 1.93	31.51 ± 3.17	0.3569
MCH (pg/cell)	32.61 ± 1.18	32.80 ± 1.06	0.6275
RDW (%)	13.4 ± 0.54	13.6 ± 1.86	0.3433

SBP, systolic blood pression; DBP, diastolic blood pression; WBC, white blood cell; RBC, red blood cell; HGB, hemoglobin; HCT, hematocrit; MCV, mean corpuscular volume; MCH, mean corpuscular hemoglobin; RDW, red cell distribution width; MCHC, mean corpuscular hemoglobin concentration. Values are presented as the mean ± SD. Student’s *t* test was used to compare differences between two groups. Values in bold indicate statistical significance.

**Table 3:** Number of individuals using each antihypertensive medication class (%).

Treatment	Number (%)
ACEI	4 (44.4)
ARB	3 (33.3)
CCB	2 (22.2)

CEI = angiotensin-converting enzyme inhibitors, ARB = angiotensin II receptor blockers, CCB = calcium channel blockers.

## Data Availability

Data are contained within the article.
